# Ultrasound assisted natural deep eutectic solvents based sustainable extraction of *Spirulina platensis* and orange peel extracts for the development of strawberry-cantaloupe based novel clean-label functional drink

**DOI:** 10.1016/j.ultsonch.2025.107357

**Published:** 2025-04-19

**Authors:** Kashmala Chaudhary, Samran Khalid, Taghrid S. Alomar, Najla AlMasoud, Sadia Ansar, Ahmed Fathy Ghazal, Abderrahmane Aït-Kaddour, Rana Muhammad Aadil

**Affiliations:** a National Institute of Food Science and Technology, University of Agriculture, Faisalabad 38000, Pakistan; b Department of Chemistry, College of Science, Princess Nourah bint Abdulrahman University, PO Box 84428, Riyadh 11671, Saudi Arabia; c Agricultural Engineering Department, Faculty of Agriculture, Suez Canal University, Ismailia 41522, Egypt; d Universit́ Clermont Auvergne, INRAE, VetAgro Sup, UMRF, 15000 Aurillac, France; e Department of Food Technology, Faculty of Agroindustrial Technology, University of Padjadjaran, Sumedang 45363 Jawa Barat, Indonesia

**Keywords:** Fruit juices blend, Natural extracts, Clean-label ingredients, Ultrasound assisted extraction, Natural deep eutectic solvents, Functional beverages

## Abstract

The consumer demand for sustainable, nutrient-rich, and clean-label beverages is growing rapidly, so there is an urgent need to meet this demand. In response, the present study introduced the new concept of clean-label functional foods and beverages. The objective of the research was to develop novel ready-to-serve clean-label functional drink formulations by blending strawberry and cantaloupe juices (35 % each), with the addition of *Spirulina platensis* and orange peel extracts as clean-label functional ingredients, either alone or in combination, at concentrations of 2.5 % and 5.0 %, without any chemical additives or preservatives. Ultrasound-assisted extraction under optimized conditions, pulsating mode (10 s on, 5 s off), power 300 W, frequency 25 kHz, extraction time 30 min, and temperature 40 °C, with natural deep eutectic solvent composed of lactic acid and choline chloride (2:1) was used to obtain *S. platensis* and orange peel extracts. This innovative method achieved the highest extract yield compared to other extraction techniques, making these extracts ideal clean-label functional ingredients due to their highly efficient, sustainable, and eco-friendly extraction process. The formulations were subsequently homogenized via ultrasonication with mild parameters (power 100 W, frequency 25 kHz, time 5 min, temperature 25 °C) to improve the overall stability of the drink. Comprehensive analysis revealed that the T6 formulation, containing 5.0 % *S. platensis* and 5.0 % orange peel extract, exhibited the most promising results across various parameters, including improved proximate composition, enriched mineral content, elevated bioactive compounds, enhanced antioxidant activity, potent antimicrobial properties, and superior sensory acceptance. By introducing and validating the concept of clean-label functional beverages, this study paves the way for future innovations that align with the evolving demands of health-conscious consumers, and environmental sustainability.

## Graphical abstract

**Figure:**
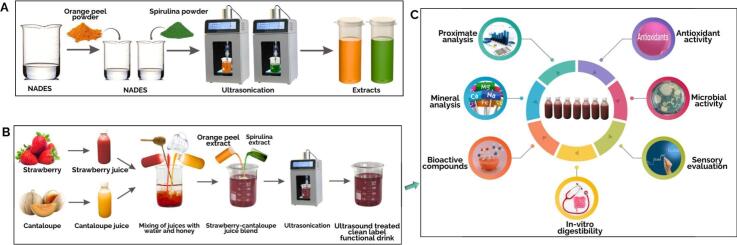

## 1 Introduction

In the current era, the food industry is facing numerous challenges such as the growing consumer awareness regarding health, nutrition, and sustainability [1,2]. Consumers are now aware of the impact of artificial additives, chemical preservatives, excessive sugar or fat consumption, and highly processed foods, driving a shift toward functional and clean-label food options [3]. Functional foods are those that incorporate various bioactive components at effective doses that extend beyond mere nutritional effects, imparting advantages in preventing and alleviating the onset of diseases [4]. Clean-label food and beverages are made with simple, natural, and recognizable ingredients, without artificial additives, synthetic chemicals, or overly processed components [5]. Functional beverages have emerged as a rapidly expanding segment within the functional food industry with key ingredients including probiotics, prebiotics, vitamins, minerals, antioxidants, herbal extracts, amino acids, and plant-based compounds known for their health-promoting properties [6,7,8].

Fruit-based functional juices have gained the most popularity due to their natural bioactive composition, rich nutrient content, potential health benefits and high sensory value [9]. In addition to single-fruit juices, fruit juice blend-based beverages have emerged as a popular alternative, offering enhanced flavor profiles and synergistic health benefits. These blends are incorporated with functional ingredients to boost their health effects making them functional beverages. This study focuses on blending strawberry and cantaloupe juices due to their complementary nutritional profiles, functional properties, and sensory appeal. Strawberries are highly valued for their flavor, aroma, and nutritional benefits and they are packed with several essential nutrients [10]. Cantaloupes, also known as muskmelons, are liked for their sweet flavor, high water content, and refreshing taste, making them a nutritious and hydrating choice [11]. While the blend of strawberry and cantaloupe juices offers a well-balanced nutritional and sensory profile, additional functional ingredients need to be added to develop a functional beverage. Microalgae are gaining significant attention in the functional food industry due to their exceptional nutritional composition and bioactive properties [12,13,14]. Among these, a noteworthy cyanobacterium is *Spirulina platensis*, also referred to as *Arthrospira platensis*. This blue-green microalga is increasingly being utilized in functional food development because of its rich nutritional profile, including high-quality proteins, essential amino acids, polyunsaturated fatty acids, and an abundance of bioactive compounds such as phycocyanin, carotenoids, and phenolics [15]. The growing concern over food waste has led to increased interest in the utilization of agro-food waste for functional food development [16,17,18]. Orange peel waste is one of the most valuable agro-food waste because it is an excellent source of flavonoids, polyphenols, pectin, essential oils, and vitamin C making them best for functional food ingredients [19,20].

The growing consumer demand for clean-label ingredients, products, and processing technologies has shifted industrial trends toward more sustainable practices. Recovering extracts from algae and food waste is a key step in utilizing them as natural ingredients for the development of functional foods and beverages. Traditional extraction methods using organic solvents have been widely employed, however, their several limitations, have highlighted the need for sustainable, eco-friendly, clean-label extraction technologies and solvents [21,22]. Among the most advanced and sustainable clean-label extraction techniques, ultrasound-assisted extraction (UAE) has gained significant attention due to its high efficiency, reduced time, and lower energy consumption [23,24]. Additionally, the use of novel green solvents, such as natural deep eutectic solvents (NADES), has emerged as an eco-friendly alternative to conventional solvents for obtaining valuable substances from various natural sources [25,26,27]. These extracts not only play as clean-label ingredients for functional food but also enhance the shelf life because of their substantial antioxidant and antimicrobial properties [28,29].

Driven by rising consumer demand for healthier food and beverages developed from sustainable clean-label sources and technologies, this study introduced a new concept focused on developing a novel ready-to-serve clean-label functional drink without any harmful additives offering dual benefits. The formulations combined strawberry and cantaloupe juices in optimized concentrations, added with *S. platensis* and orange peel extracts as clean-label functional ingredients obtained through the sustainable UAE with NADES. Comprehensive testing was conducted to evaluate the drink’s nutritional composition, bioactive compounds, in-vitro digestion, antimicrobial properties, and sensory attributes. To the best of our knowledge, this is the first study to develop a clean-label functional drink from blended fruit juices with the addition of composite extracts derived from both algal and waste materials using UAE with NADES, creating a synergistic combination that enhances the nutritional profile. The study’s primary focus was to utilize sustainable ingredients, processes, and technologies, aligning with global efforts to promote environmentally friendly innovation.

## 2 Materials and Methodology

### 2.1 Raw materials

Fully mature, fresh, and ripe strawberries (*Fragaria × ananassa*, cultivar: Chandler) were sourced from farms in Kasur, Pakistan, at commercial maturity, characterized by a fully red color, firm texture, and an optimal sugar-to-acid ratio for harvest. Likewise, fully ripe cantaloupes (*Cucumis melo*, cultivar: Hales Best) were obtained from farms in Bahawalpur, Pakistan, at physiological maturity, identified by uniform netting, yellowish skin color, and a distinctive ripe aroma. All the fruits were undamaged and disease-free specimens of uniform color, size, and ripening stage. Orange peel powder and *S. platensis* powder were bought online from Daraz.pk (Pakistan). Honey was brought from a local village in Faisalabad, Pakistan. All chemicals used in this study were of lab grade and were purchased from Sigma-Aldrich (Merck KGaA, Darmstadt, Germany).

### 2.2 Preparation of NADES and clean-label extracts

The NADES used for UAE was prepared by following the commonly used method of heating and stirring. Lactic acid, acting as a hydrogen bond donor, and choline chloride, acting as a hydrogen bond acceptor, were mixed in (2:1) concentration and heated with continuous stirring at 80 °C until a clear, homogeneous liquid was obtained. The viscosity of the liquid was adjusted by adding 30 % water. The UAE process was carried out by following the procedure used by Airouyuwa et al. [25] with some modifications. The extraction suspensions were prepared by separately mixing *S. platensis* and orange peel powder in a NADES. The suspensions, with a sample-to-solvent ratio of 1:30 g/mL, were pre-soaked for 30 min to facilitate cell disruption. UAE was conducted using a probe-type ultrasonicator (SCIENTZ-750F, Ningbo, China) operating at a pulsating mode (10 s on: 5 s off) with a power of 300 W and a frequency of 25 kHz for 30 min. The temperature was maintained at approximately 40 °C by placing the flask in an ice bath to prevent heat build-up. To obtain extracts without ultrasonication (US) using only NADES, the sample and NADES were mixed in a 1:30 g/mL and heated at 60 °C for 3 h. To compare the extraction efficiency of NADES with organic solvent, ethanol was used in the above UAE procedure instead of NADES. For the extraction without US, the Soxhlet apparatus was used in which 10 g samples of *S. platensis* and orange peel powder were mixed in 250 mL of 70 % ethanol, and extraction was performed at 50 °C for 5 h. After the processes, the suspensions were centrifuged at 10,000 rpm for 10 min using a refrigerated benchtop centrifuge (416KS, Sigma, Osterode, Germany), and the supernatants were collected for analysis and further use as clean-label ingredients.

### 2.3 Characterization of clean-label extracts

The standard procedures of AOAC. [30] were followed with slight modifications to determine the composition of the extracts. Proteins were analyzed using the Kjeldahl method, lipids by Soxhlet extraction, total phenolic content (TPC) via the Folin-Ciocalteu assay, and total flavonoid content (TFC) using the aluminum chloride method. Antioxidant activity was assessed through DPPH free radical scavenging activity, while chlorophyll *a*, chlorophyll *b*, and carotenoids were quantified using spectrophotometric methods. Carotenoids were also analyzed using HPLC for precise quantification.

### 2.4 Development of functional drink formulations

**Fig. 1:**
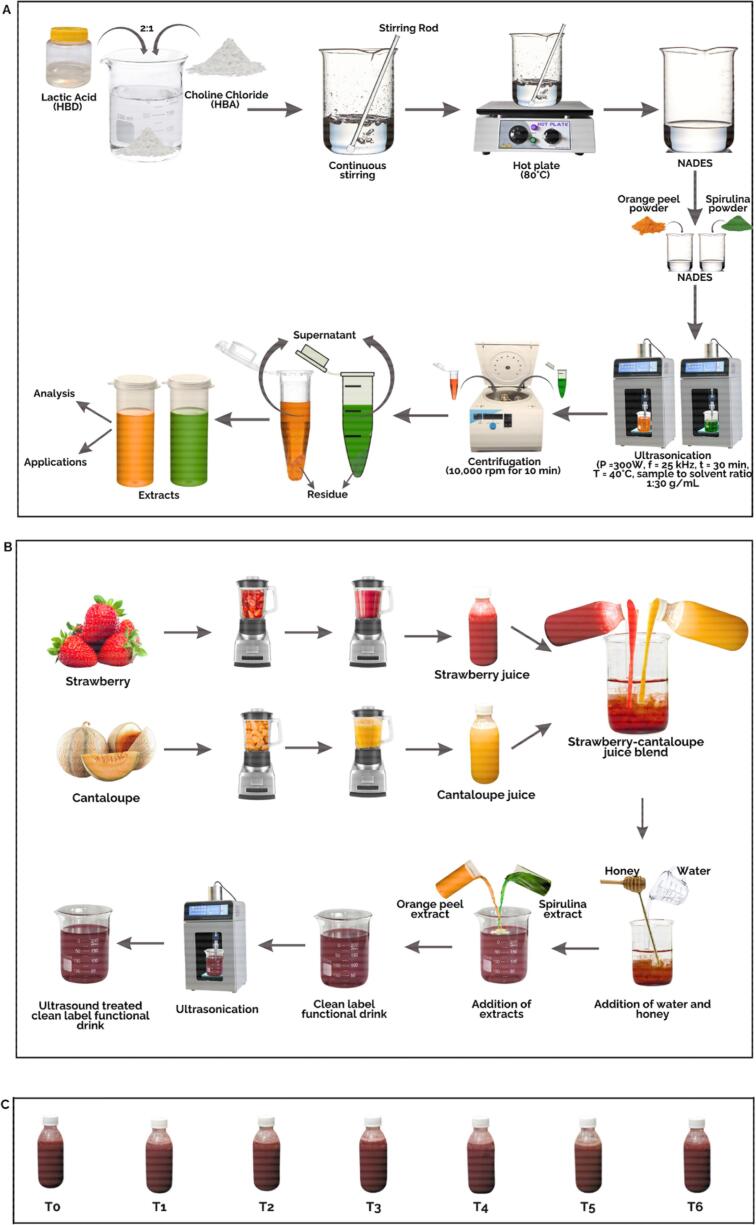
Schematic illustration of the step-by-step experimental process for the development of a novel clean-label functional drink. (A) Illustration of the recovery of extracts from *Spirulina platensis* and orange peel powder using Ultrasound assisted extraction with natural deep eutectic solvents, highlighting key steps and parameters, (B) Development of clean-label functional drink formulations by blending strawberry and cantaloupe juices with the addition of varying concentrations (2.5% and 5%) of *Spirulina platensis* and orange peel extracts along with the application of Ultrasonication for homogenization, (C) Clean-label functional drink formulations T0, T1, T2, T3, T4, T5, and T6.

The fruits were sorted and washed to remove any contaminants. The inedible parts of the strawberry, as well as the peel and seeds of the cantaloupe, were removed. The fruits were then cut to reduce their size, and the pulps were blended using a high-speed power blender (HR3752/00, Amsterdam, Netherlands). The blended pulp was filtered through a double-layered muslin cloth to separate the fine juice from any residual fibers or solids. The juices were blended in different ratios to determine the optimal combination based on consistency, flavor, and overall sensory acceptability. After evaluation, a ratio of 35 % strawberry and 35 % cantaloupe was selected, as it provided an ideal balance of texture, taste, and appearance. To improve the nutritional value and make the drink suitable for people of all ages, 5 % honey was added as a sweetener, replacing sugar, thus improving the flavor. Additionally, 0.5 % pectin was added as a stabilizer to the blended juices. Subsequently, a total of six treatments (T1, T2, T3, T4, T5 and T6), each 100 mL, of ready-to-serve strawberry and cantaloupe juice blends were prepared as novel clean-label functional drinks by incorporating varying amounts of UAE-NADES obtained extracts, as outlined in Table 1 keeping only juice blend as control (T0). The concentration of juice and honey remained consistent across all formulations, while the concentrations of water and extracts varied. To improve texture, ensure stability, enhance uniformity, and prevent phase separation, the functional drink formulations were homogenized with US that was applied using mild parameters using a probe-type ultrasonicator at a power of 100 W and a frequency of 25 kHz for 5 min along with maintaining the temperature at 25 °C by placing the flask in an ice bath. The UAE-NADES extraction of *S. platensis* and orange peel powder extracts, along with the development of clean-label functional drink formulations, is depicted in Fig. 1.

**Table 1:** Treatments of ready-to-serve strawberry and cantaloupe juice blends based on clean-label functional drinks.

**Samples**	**Strawberry Juice** **(%)**	**Cantaloupe Juice** **(%)**	**Honey (%)**	**Water** **(%)**	***Spirulina platensis* extract** **(%)**	**Orange peel extract (%)**
T0	35	35	5	25.0	−--	−--
T1	35	35	5	22.5	2.5	−--
T2	35	35	5	22.5	−--	2.5
T3	35	35	5	20.0	2.5	2.5
T4	35	35	5	20.0	5.0	−--
T5	35	35	5	20.0	−--	5.0
T6	35	35	5	15.0	5.0	5.0

### 2.5 Analysis of functional drink formulations

#### 2.5.1 Proximate composition

The protein content of formulations was determined using the Kjeldahl method, while lipid content was assessed through the Soxhlet extraction method. Fiber was measured using the Total Dietary Fiber by Enzymatic-Gravimetric Method, and carbohydrate content was calculated by difference. All analyses were conducted following guidelines of AOAC. [30].

#### 2.5.2 Minerals profile

By following the procedure of Hassanzadeh et al. [4], the mineral profile (potassium (K), magnesium (Mg), calcium (Ca), iron (Fe), manganese (Mn), zinc (Zn), and sodium (Na)) of formulations was assessed. The wet digestion method was used, which involved placing a 5 mL sample of drink in a 100 mL beaker containing 10 mL of nitric acid (65 %). It was covered with a watch glass and then the mixture was heated at 120 °C on a hot plate, and digestion was continued until the fumes emission stopped and the solution became colorless. After cooling, the double distilled water was added to the solution taken in a volumetric flask, and afterwards digested samples were checked in an atomic absorption spectrometer (PerkinElmer AAnalyst™ 700, Waltham, USA).

#### 2.5.3 Determination of pH, total soluble solids (TSS), and titratable acidity (TA)

The pH of the formulations was measured using a digital pH meter which was calibrated with buffer solutions at pH 4.0 and pH 7.0. The TSS was determined using a digital refractometer. The TA was analyzed using the acid-base titration method. 10 mL sample was diluted with 50 mL of distilled water and titrated against 0.1 N NaOH using phenolphthalein as an indicator until a persistent pink color appeared.

#### 2.5.4 Total phenolic content (TPC) and total flavonoid content (TFC)

TPC and TFC of the formulations were determined by the procedure used by Ebrahimi & Rastegar. [31]. The sample was extracted using 80 % methanol, and the resulting homogenate was centrifuged at 5,000 rpm for 10 min to collect the supernatant for further analysis. To determine TPC, a reaction mixture was prepared by combining 0.5 mL of the methanolic extract, 1.5 mL of 5 % sodium carbonate, and 1.5 mL of diluted Folin-Ciocalteu reagent. The mixture was incubated for 90 min, and its absorbance was measured at 750 nm using a spectrophotometer. For TFC determination, a reaction mixture consisting of 0.5 mL of the methanolic extract, 0.1 mL of 10 % aluminum chloride (AlCl_3_), and 0.1 mL of 1 M potassium acetate solution was prepared. After 30 min incubation period, the absorbance was measured at 415 nm.

#### 2.5.5 Total carotene content (TCC)

The UV-spectrophotometer (Shimadzu UV-2600, Shimadzu Corporation, City, Japan) was used to assess the total carotene content by following the method of Abidoye et al. [32]. The solvent mixture (hexane-acetonitrile-ethanol, 50:25:25) was used to extract carotenoids from the sample. The sample and solvent mixture were combined in a 1:20 ratio, stirred for 15 min, and homogenized for 10 min. After homogenization, 5 mL of distilled water was added, and the mixture was allowed to rest for 10 min to facilitate phase separation. The upper hexane layer, containing the carotenoids, was carefully collected for analysis. Hexane was used as a blank, and the absorbance was measured at 450 nm using the UV-spectrophotometer.

#### 2.5.6 Antioxidant activity

The antioxidant activity of the formulations was measured by conducting three assays. The method of Agunbiade et al. [33] was used for conducting the DPPH (1,1-diphenyl-2-picrylhydrazyl) radical scavenging assay by preparing a reaction mixture by mixing 400 μL of the sample's methanolic extract with 200 μL of distilled water and 600 μL of 0.1 mM DPPH solution. The mixture was incubated in the dark at room temperature for 30 min to ensure the reaction reached completion. Following incubation, the absorbance was measured at 517 nm using a spectrophotometer, and the antioxidant activity was expressed as the percentage of DPPH inhibition. The ABTS [2,2′-azino-bis(3-ethylbenzothiazoline-6-sulphonic acid)] assay was performed by following the procdure of Bendaali et al. [34], by preparing 7 mM ABTS mixed with 2.5 mM potassium persulfate and incubating the solution in the dark for 16 h. The solution was diluted with ethanol to adjust absorbance to 0.70 ± 0.02 at 734 nm. Subsequently, 30 µL of the sample was mixed with 3 mL of the ABTS solution and allowed to react in the dark for 6 min. The absorbance of the mixture was measured at 734 nm using a spectrophotometer. Ferric Reducing Antioxidant Power (FRAP) assay was employed to evaluate the sample's antioxidant capacity by following the method of Ashfaq et al. [35]. FRAP solution was prepared by mixing 25 mL of 300 mM acetate buffer, 2.5 mL of 10 mM TPTZ, and 2.5 mL of 20 mM FeCl_3_·6H_2_O. The sample was combined with the prepared FRAP solution and incubated for 10 min in the dark to facilitate the reaction. Following incubation, the absorbance was measured at 593 nm using a spectrophotometer, and the antioxidant activity was calculated based on a linear standard curve of FeSO_4_ and expressed as μM Fe^2+^/mL.

#### 2.5.7 Reducing, non-reducing and total sugars

The reducing, non-reducing and total sugars of formulations were determined by the methods followed by Saleem et al. [36] with some modifications. Fehling’s test was utilized to quantify reducing sugars in drink samples. Initially, the sample was treated with 5 % lead acetate (1:1 v/v) to remove impurities and potassium oxalate to eliminate excess lead. After filtration, the solution underwent titration against Fehling's solution, with 1 % methylene blue serving as an indicator. The endpoint was identified by the disappearance of the blue color, indicating a complete reduction of Cu2^+^ to Cu_2_O. Non-reducing sugars were hydrolyzed into reducing sugars by treating the sample with 1 M hydrochloric acid and incubating it at 70 °C for 1 h. The solution was then neutralized with 0.1 N NaOH and subsequently titrated against Fehling's solution. To determine total sugars, the sample was hydrolyzed using 2.5 M HCl for 2.5 h, followed by neutralization with Na_2_CO_3_. The homogenate was centrifuged at 6000 rpm for 10 min, and 10 μL of the supernatant was diluted with 1 mL of distilled water. Then, 1 mL of 5 % crystalline phenol and 5 mL of concentrated H_2_SO_4_ (96 %) were added. The mixture was vortexed and incubated at 25 °C for 20 min. Absorbance was measured at 420 nm against a reagent blank, and the sugar concentration was determined using a standard curve of D-glucose.

#### 2.5.8 Ascorbic acid

The indophenol titration method was used to determine the ascorbic acid content, following the procedure outlined by Chettri et al. [37]. A 5 mL sample was mixed with 5 mL of 0.5 % oxalic acid solution to prevent oxidation and filtered through the Whatman No. 1 paper. The filtrate was titrated against freshly prepared 2,6-dichlorophenolindophenol (DCPIP) solution until a persistent pink endpoint appeared.

#### 2.5.9 Color

The portable colorimeter (Konica Minolta CR-5, Tokyo, Japan) was used for the color analysis of the formulations. The coordinates L* (lightness), a* (green (−) to red (+)) and b* (blue (−) to yellow (+)) were assessed.

#### 2.5.10 Viscosity, cloud value, and non-enzymatic browning

The viscosity of the formulations was measured using a Brookfield viscometer (Model DV2T, Middleborough, USA) equipped with an LV-2 spindle. A 50 mL sample was poured into the beaker, and viscosity was evaluated at a rotation speed of 100 rpm at 25 °C. The measurement was taken for 30 s, or until the reading stabilized. For assessing the cloud value, a 5 mL sample was centrifuged at 3500 × g for 15 min. The absorbance of the supernatant was then measured at 660 nm using a spectrophotometer, with distilled water used as the blank. For assessing non-enzymatic browning, the sample was centrifuged at 12,500 × g for 10 min. The absorbance of the supernatant was then measured at 420 nm using a spectrophotometer.

#### 2.5.11 In-vitro digestibility

In-vitro digestibility of the formulations was assessed by using the method followed by Fradinho et al. [38]. A 1 mL sample from each formulation was mixed with 25 mL of phosphate buffer (0.1 M, pH 6.0) in a 250 mL conical flask. The pH was adjusted to 2.0 using 10 mL of 0.2 M HCl, and 3 mL of a freshly prepared pepsin solution (30 mg of porcine pepsin, 0.8 FIP-U/mg) dissolved in buffer was added. The mixture was incubated at 39 °C for 3 h with constant agitation. After gastric digestion, 10 mL of phosphate buffer and 5 mL of 0.6 M NaOH were added to adjust the pH to 6.8. A pancreatin solution (10 mL, dissolved in phosphate buffer, 500 mg of porcine pancreatin, 42,362 FIP-U/g) was then introduced, and the samples were incubated at 39 °C for an additional 9 h with continuous agitation. The pH was checked and adjusted if necessary during incubation. A reagent blank without a sample was prepared for reference. After digestion, undigested residues were collected by centrifugation, washed with deionized water, and dried at 55 °C until a constant weight was achieved. The in-vitro digestibility (%) was calculated as the difference between the initial dry biomass and the undigested dry biomass (after blank correction), divided by the initial dry biomass, and multiplied by 100.

#### 2.5.12 Microbial analysis

The method used for determining the total plate, total yeast and total mold count, of all the samples was described in the study of Mehra et al. [39]. For the total plate count, 0.1 mL from serial dilutions was transferred onto plate count agar in a petri dish and incubated at 37 °C for 24 h. For the total yeast and mold count, 0.1 mL of the sample from serial dilutions was plated on potato dextrose agar. The yeast plates were incubated at 25–28 °C for 3–5 days, while the mold plates were incubated at 25–28 °C for 5–7 days to allow visible colony formation.

#### 2.5.13 Sensory evaluation

All the formulations of the prepared functional drink formulations were evaluated for their sensory attributes using a hedonic scale (where 1 represented extremely dislike and 9 represented extremely like). Fifteen trained panelists, aged between 30–50 years, were selected based on their prior experience with sensory evaluation in food studies, regular participation in taste panels, and completion of sensory training sessions. Each judge provided informed consent after receiving a clear explanation of the study's purpose and significance. Participation was entirely voluntary, and judges had the freedom to withdraw at any point without facing any consequences. The evaluation was conducted in a quiet place with adequate lighting and a moderate temperature (22 ± 2°C). The sensory index system included appearance, aroma, flavor, mouthfeel, and overall acceptability. The judges performed the sensory analysis based on the scoring system. Judges were given room-temperature water and plain crackers to cleanse their palates between samples, with a 2-min break provided after each evaluation to prevent sensory fatigue.

### 2.6 Statistical analysis

Statistical analysis was performed using the SPSS program (IBM SPSS Statistics V25.0, Chicago, USA). One-way analysis of variance (ANOVA) with the Tukey pairwise comparison test was used to determine the significant differences between mean values at a significance level of P < 0.05. The results are reported as means ± standard deviations. Principal component analysis (PCA) was used to analyze the relationship and differences between the novel functional drink formulations with different treatments (T0, T1, T2, T3, T4, T5 and T6) using Simca Statistical Software (Version 14.1, Simca, MKS Umetrics AB).

## 3 Results and Discussion

### 3.1 Characterization of *S. platensis* and orange peel extracts

The active ingredients obtained from natural materials play a significant role in the development of functional foods and beverages because of their rich functional composition [40]. The rich functional composition of *S. platensis* and orange peel extracts obtained in our research (Table 2) confirms this ongoing narrative and demonstrates their potential for use in the development of functional food products.

**Table 2:** Composition of *S. platensis* and orange peel extracts obtained using different methods.

**Extraction method**	**Protein (mg/g)**	**Lipids (mg/g)**	**Total phenolic content** **(mg GAE/g)**	**Total flavonoid content** **(mg QE/g)**	**DPPH free radical assay (%)**	**Chlorophyll *a* (mg/g)**	**Chlorophyll *b* (mg/g)**	**Carotenoids (mg/g)**
***Spirulina platensis* extracts**								
UAE with NADES	655.40 ± 3.68^a^	74.91 ± 2.63^a^	33.46 ± 2.23^a^	1.85 ± 0.08^a^	69.06 ± 0.86^a^	31.76 ± 0.12^a^	23.78 ± 0.06^a^	6.93 ± 0.04^a^
NADES	435.32 ± 2.94^c^	34.83 ± 3.13^c^	17.53 ± 0.63^c^	0.33 ± 0.05^c^	48.69 ± 0.35^c^	17.23 ± 0.12^c^	13.27 ± 0.06^c^	4.77 ± 0.04^c^
UAE with ethanol	504.35 ± 2.94^b^	55.28 ± 2.57^b^	21.93 ± 1.50^b^	0.90 ± 0.05^b^	63.06 ± 0.83^b^	24.36 ± 0.06^b^	18.37 ± 0.05^b^	6.07 ± 0.05^b^
Ethanol	380.02 ± 3.51^d^	35.49 ± 1.99^c^	13.58 ± 2.22^d^	0.13 ± 0.03^d^	32.60 ± 0.36^d^	7.83 ± 0.05^d^	8.22 ± 0.05^d^	2.55 ± 0.03^d^
**Orange peel extracts**								
UAE with NADES	7.81 ± 4.38^a^	5.58 ± 2.43^a^	36.51 ± 2.38^a^	28.66 ± 1.21^a^	74.04 ± 0.58^a^	ND	ND	1.77 ± 0.05^a^
NADES	2.95 ± 3.98^c^	4.17 ± 1.61^c^	19.61 ± 1.86^c^	18.92 ± 0.50^c^	51.44 ± 0.56^c^	ND	ND	0.63 ± 0.02^c^
UAE with ethanol	5.17 ± 3.82^b^	4.72 ± 3.04^b^	33.06 ± 1.34^b^	21.82 ± 0.56^b^	61.63 ± 0.41^b^	ND	ND	1.35 ± 0.03^b^
Ethanol	1.05 ± 3.23^d^	3.13 ± 2.50^d^	23.61 ± 2.33^d^	13.75 ± 0.47^d^	49.85 ± 0.50^d^	ND	ND	0.22 ± 0.04^d^

(ND; Not detected) Data are expressed as means ± SD and values with different superscript letters in a column of each group differ significantly (P < 0.05).

*S. platensis* extracts obtained via UAE-NADES showed the highest protein content (655.40 ± 3.68 mg/g) and lipid content (74.91 ± 2.63 mg/g) compared to other extraction methods that yielded protein values ranging from 504.35 ± 2.94 mg/g to 380.02 ± 3.51 mg/g and lipids 55.28 ± 2.57 mg/g to 34.83 ± 3.13, highlighting the significance of the extraction method and solvent in macromolecules extraction. A similar trend in extraction efficiency was observed for orange peel extracts, with the highest yield of protein (7.81 ± 4.38 mg/g) and lipids (5.58 ± 2.43 mg/g) achieved through UAE with NADES. And the protein and lipid contents obtained through other methods ranged from 5.17 ± 3.82 mg/g to 1.05 ± 3.23 mg/g for proteins, and 4.72 ± 3.04 mg/g to 3.13 ± 2.50 mg/g for lipids across all other extraction methods. The study by Zhou et al. [41] and Singh et al. [42] aligns with our study, as they also demonstrated the high efficiency of UAE (high cell disruption) with these novel solvents in extracting proteins and lipids from various algal and plant-based sources, outperforming conventional organic solvents-based extraction.

In the case of TPC and TFC, the orange peel extracts had higher values of TPC (36.51 ± 2.38–19.61 ± 1.86 mg GAE/g) and TFC (28.66 ± 1.21–13.75 ± 0.47 mg QE/g) obtained using the four extraction methods (UAE with NADES > UAE with ethanol > NADES > ethanol) as compared to *S. platensis* extracts, which had TPC (33.46 ± 2.23–13.58 ± 2.22 mg GAE/g) and TFC (1.85 ± 0.08–0.13 ± 0.03 mg QE/g). This also explains why the antioxidative potential of orange peel extracts (74.04–49.85 %) was higher than that of *S. platensis* (69.06–32.6 %). Moreover, the high values obtained with UAE-NADES compared to other methods highlight the efficiency of UAE with NADES in stabilizing phenolics and flavonoids while enhancing their release from cells due to high disruption [43]. The study by Airouyuwa et al. [25] confirms our results, where they obtained three times higher TPC, TFC, and antioxidant activity from date seeds using UAE-NADES compared to UAE-ethanol. Our findings also align well with the research of Zurob et al. [44] where authors obtained 87 ppm of hydroxytyrosol from olive leaves by lactic acid:glucose (5:1) as compared to 30 ppm obtained using ethanol (50 %).

The *S. platensis* extracts had chlorophyll *a* content (31.76 ± 0.12 mg/g − 7.83 ± 0.05 mg/g), chlorophyll *b* (23.78 ± 0.06 mg/g − 8.2 ± 0.12 mg/g), and carotenoids (6.93 ± 0.04 mg/g − 2.55 ± 0.03 mg/g) obtained through four extraction methods. This demonstrates enhanced pigment stability and protection against degradation. In orange peel extracts, no chlorophyll *a* or b was detected due to the absence of these pigments in citrus peel. However, the carotenoid content obtained ranged from 1.77 ± 0.05 mg/g − 0.22 ± 0.04 mg/g. Our results align with the findings of Martins et al. [45], which demonstrated the efficiency of UAE-NADES in pigment extraction from *S. platensis*, and Viñas-Ospino et al. [46], where 1.635 ± 0.011 mg/g of carotenoids was extracted from orange peel by UAE with NADES menthol:camphor. This is comparable to our result of 1.77 ± 0.05 mg/g by UAE with lactic acid:choline chloride.

So, the composition of extracts revealed significant variation depending on the extraction method, as shown in Table 2. UAE was chosen because of its proven extraction efficiency in extracting sensitive substances. US generates cavitation, which produces microbubbles that collapse violently, creating localized high pressure and temperature. This mechanical effect disrupts cell walls, enhances mass transfer, and facilitates the release of bioactive compounds from plant matrices. As a result, it significantly improves extraction efficiency compared to conventional methods. The eco-friendly NADES based on lactic acid and choline chloride was chosen based on our extensive literature review, where research confirmed its high extraction efficiency [47]. The molar ratio, viscosity, temperature, pH, extraction duration, sample-to-solvent ratio, polarity, solubility and particle size of samples were carefully optimized, resulting in the highest yield. NADES, composed of natural and biodegradable components offer superior solvent properties for extracting valuable substances even from tough materials [25]. Properly optimizing the molar ratio, viscosity, temperature, pH, extraction duration, sample-to-solvent ratio, polarity, solubility, sample particle size, and ability of the solvent to interact with target molecules through hydrogen bonding and van der Waals forces make it highly effective in dissolving a wide range of substances. It can be seen in Table 2 that UAE with NADES exhibited superior extraction efficiency over other extraction methods across all measured parameters, reinforcing its role as the most sustainable and effective advanced extraction method. Therefore, these clean-label extracts were chosen for subsequent addition to the juice blend for functional drink formulations. It is clearly indicated that extracts obtained from algal and waste materials using different extraction methods will significantly affect the properties of the functional drink, as the extract composition depends on the extraction method. Afterwards, the highest yield of all the components was obtained by UAE with ethanol, as ethanol is also a widely used and efficient organic solvent, followed by NADES alone and then ethanol alone in heat-based simple extraction methods. Moreover, our overall results for *S. platensis* and orange peel extract composition are somewhat higher or similar in some cases compared to the detailed review and research studies of Seghiri et al. [48], AlFadhly et al. [49], and Oliveira et al. [2] who conducted research on a comprehensive assessment of *S. platensis*, and Ortiz-Sanchez et al. [50], who extensively evaluated orange peel. This rich nutritional composition of these extracts makes them very effective candidates in the development of functional food products.

### 3.2 Proximate composition of formulations

Strawberry and cantaloupe juices are naturally rich in various essential nutrients, and blending two different juices enhances their overall nutritional profile by compensating for each other's limitations of any nutrients. This synergy not only boosts the availability of diverse nutrients but also improves sensory attributes such as appearance, aroma, flavor, texture, and mouthfeel ultimately increasing consumer preference and acceptability. Moreover, the incorporation of active clean-label extracts significantly enhances the drink's nutritional value, making it a highly nutrient-rich clean-label functional drink. The proximate composition (protein, lipids, fiber, and carbohydrates) of the formulations is elaborated in Table 3. The protein values showed variation across all formulations (T0, T1, T2, T3, T4, T5,T6) (Table 1), however, the values were substantially higher in the formulations containing *S. platensis* extract due to its high protein content. T6 exhibited the highest protein value of 1.97 ± 0.05 g/100 mL, as it contained 5 % of both extracts (*S. platensis* and orange peel), followed by T4, which had 1.88 ± 0.05 g/100 mL with 5 % *S. platensis* extract only. T0, which contained no extracts, had the lowest protein value of 0.07 ± 0.03 g/100 mL, as fruits are not rich sources of protein. Meanwhile, T2 with 2.5 % and T4 with 5 % orange peel extract had only 0.14 ± 0.03 g/100 mL and 0.19 ± 0.04 g/100 mL of protein, respectively. This demonstrates the ability of *S. platensis* extract to enhance the protein content of functional drink formulations as compared to control (T0). The study by Oliveira et al. [2], Barkallah et al. [10], and Batista et al. [11] also reported an increase in the protein content of chocolate milk drinks, yogurt and cookies with the addition of *S. platensis* confirming its high protein content. Moreover, the study by El-Nawasany et al. [51] also showed a slight increase in protein with the addition of orange peel powder to the yogurt.

**Table 3:** Proximate and minerals composition of clean-label functional drink formulations.

**Treatments**	**Protein (g/100 mL)**	**Lipids (g/100 mL)**	**Fibre (g/100 mL)**	**Carbohydrates (g/100 mL)**	**Potassium (mg/100 mL)**	**Magnesium (mg/100 mL)**	**Calcium (mg/100 mL)**	**Iron (mg/100 mL)**	**Manganese (mg/100 mL)**	**Zinc** **(mg/100 mL)**	**Sodium (mg/100 mL)**
**T0**	0.07 ± 0.03^c^	0.03 ± 0.02^c^	0.24 ± 0.02^d^	8.15 ± 0.05^d^	45.67 ± 2.21^g^	8.17 ± 0.24^g^	9.52 ± 0.38^g^	0.14 ± 0.05^f^	0.07 ± 0.02^c^	0.05 ± 0.01^d^	1.24 ± 0.04^d^
**T1**	1.02 ± 0.06^b^	0.16 ± 0.03^b^	0.28 ± 0.02^d^	8.23 ± 0.05^bcd^	65.78 ± 0.73^d^	17.88 ± 0.45^d^	14.50 ± 0.41^f^	0.27 ± 0.01^d^	0.14 ± 0.03^bc^	0.13 ± 0.02^bc^	3.54 ± 0.39^c^
**T2**	0.11 ± 0.03^c^	0.08 ± 0.02^c^	0.85 ± 0.03^b^	8.33 ± 0.04^ab^	52.46 ± 1.09^f^	11.78 ± 0.55^f^	17.78 ± 0.38^e^	0.22 ± 0.02^e^	0.07 ± 0.02^c^	0.07 ± 0.01^cd^	1.49 ± 0.08^d^
**T3**	1.11 ± 0.04^b^	0.18 ± 0.03^b^	0.93 ± 0.05^b^	8.27 ± 0.03^bc^	94.11 ± 0.80^b^	22.76 ± 0.40^c^	21.39 ± 0.42^c^	0.42 ± 0.04^b^	0.18 ± 0.03^b^	0.19 ± 0.03^b^	7.28 ± 0.23^b^
**T4**	1.88 ± 0.05^a^	0.32 ± 0.05 ^a^	0.40 ± 0.03^c^	8.18 ± 0.03^cd^	85.70 ± 0.32^c^	25.62 ± 0.41^b^	19.52 ± 0.36^d^	0.43 ± 0.03^b^	0.19 ± 0.05^b^	0.17 ± 0.03^b^	6.73 ± 0.27^b^
**T5**	0.14 ± 0.04^c^	0.09 ± 0.02^c^	1.48 ± 0.04^a^	8.31 ± 0.04^ab^	56.75 ± 0.28^e^	14.71 ± 0.34^e^	25.56 ± 0.32^b^	0.31 ± 0.02^c^	0.14 ± 0.03^bc^	0.06 ± 0.02^cd^	1.74 ± 0.12^d^
**T6**	1.97 ± 0.05^a^	0.35 ± 0.03^a^	1.58 ± 0.05^a^	8.40 ± 0.04^a^	141.81 ± 0.56^a^	35.72 ± 0.29^a^	33.45 ± 0.26^a^	0.68 ± 0.01^a^	0.32 ± 0.04^a^	0.33 ± 0.04^a^	12.37 ± 0.36^a^

Data are expressed as means ± SD. Values with different superscript letters in each column are significantly different (P < 0.05).

It can be seen from Table 3 that there was a slight difference in lipid content values among all formulations, with no statistically significant difference in some cases. This is because the blended fruits and extracts have very low lipid content. The lipid values for all formulations ranged from 0.05 ± 0.02 g/100 mL in T0 to 0.35 ± 0.03 g/100 mL in T6. The formulations containing *S. platensis* extract (T1 and T4 samples) had slightly higher lipid values than those containing orange peel extracts (T2 and T5 samples), as *S. platensis* has a relatively higher lipid content compared to the negligible lipid values of orange peel extracts. A low lipid content of less than 1 % is generally desirable in fruits and vegetables-based functional drinks, as it prevents an oily texture and maintains a light refreshing taste. In line with our findings, the study conducted by Nakib et al. [52] showed that the lipid content of the biscuits slightly increased with the addition of *S. platensis*, Barkallah et al. [10] found only a 0.2 % increase in fat from yogurt by the addition of *S. platensis*, on the contrary Oliveira et al. [2] demonstrated the decrease in lipid content of chocolate milk drinks with the increasing concentration of microencapsulated *S. platensis*. The findings of another study also contrasted with our results of a small increase of lipids in the drink with the addition of orange peel extract. Jahromi et al. [53] showed that the lipid content of *Chlorella Vulgaris* increased from 24 g/100 g to 31.67 g/100 g by the addition of orange peel.

There were significantly higher fiber content values in the formulations containing orange peel extracts, as microalgae are naturally low in fiber, as evident in Table 3. The fiber content increased from 0.24 ± 0.02 g/100 mL in T0 to 1.58 ± 0.05 g/100 mL in T6, which contained 5 % of both extracts. T5 showed a value of 1.48 ± 0.04 g/100 mL, slightly lower than T6, as it also contained 5 % orange peel extract. A similar pattern was observed in T3 and T4. This could be attributed to the high fiber content of fruit and vegetable waste materials, which significantly contribute to the overall fiber content of the formulations. A related study, Rezvani & Goli [54], also reported an increase in fiber content in a milk-based functional drink supplemented with carrot pomace. Elkot et al. [55] and Batista et al. [13] have demonstrated a slight increase in fiber content with the addition of *S. platensis* in fermented whey-based beverages and cookies.

The slight increase in carbohydrate content from 8.15 ± 0.05 g/100 mL in T0 to 8.40 ± 0.04 g/100 mL in T6 suggests that the incorporation of orange peel extract contributed to the overall carbohydrate levels. Formulations containing *S. platensis* extract exhibited relatively lower carbohydrate content compared to those with orange peel extracts, aligning with the findings of Oliveira et al. [2] and Batista et al. [13] where the authors observed a nonsignificant increase in carbohydrate content upon adding *S. platensis* to chocolate milk and cookies. However, a more pronounced increase in carbohydrate content was noted when waste materials were introduced, as demonstrated in Rezvani & Goli [54] research.

### 3.3 Minerals composition of formulations

The results demonstrated that the incorporation of *S. platensis* and orange peel extracts into the juice blend significantly enhanced the mineral content compared to the control (T0), as presented in Table 3. The formulations containing *S. platensis* extracts, either alone or in combination, exhibited higher concentrations of all minerals except calcium. The formulations containing orange peel extracts, either alone or in combination, had the highest calcium levels. The inclusion of algal and citrus waste extracts in the juice blends led to a threefold increase in potassium (45.67 to 141.81), a fourfold increase in magnesium (8.17 to 35.72), a threefold increase in calcium (9.52 to 33.45), a fivefold increase in iron (0.14 to 0.68), a fivefold increase in manganese (0.07 to 0.32), a sixfold increase in zinc (0.05 to 0.33), and a tenfold increase in sodium (1.24 to 12.37). This increase in minerals-related findings aligns with those from the study of Elkot et al. [55], which reported an increase in the all minerals content of whey-based sports beverages upon fortification with *S. platensis*. Similarly, Hassanzadeh et al. [4] demonstrated a comparable increase in mineral content with the addition of *S. platensis* algae in pear-cantaloupe juice. Barkallah et al. [10] reported an increase in calcium, iron and magnesium in yogurt by *S. platensis*. Furthermore, the study El-Beltagi et al. [57] also reported a similar enhancement in mineral content when cakes were enriched with prickly pear waste.

### 3.4 pH, total soluble solids (TSS), and titratable acidity (TA) of formulations

**Fig. 2:**
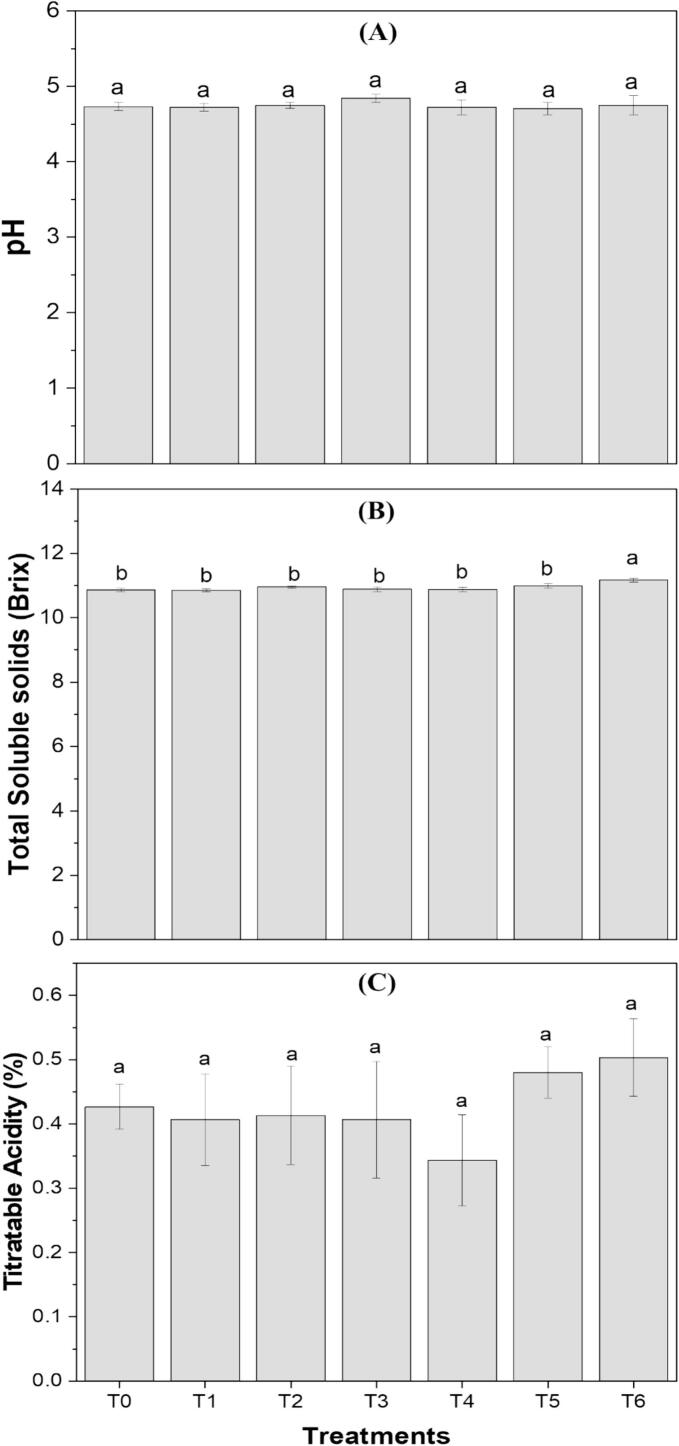
(A) pH, (B) Total soluble solids, and (C) Titratable acidity of clean-label functional drink formulations (T1, T2, T3, T4, T5, T6). Treatments with different letters above the bars indicate significant differences (P < 0.05) between the formulations.

pH, TSS, and TA are key parameters influencing the taste, stability, nutritional balance, and consumer acceptability of functional drinks. The results of pH, TSS, and TA of the clean-label functional drink formulations are presented in Fig. 2. There were no statistically significant differences in the values of pH, TSS, and TA among the different formulations. The pH of the control sample was 4.73, while the lowest value was observed in T5 (4.70). T6, which contained both extracts, showed a pH value of 4.74. The lower pH in T5 could be attributed to the presence of orange peel extract, which contains acids such as citric and ascorbic acid. In comparison, the addition of the algal extract may have contributed to the higher pH in T6 (Fig. 2 **(A)**). TSS ranged from 10.86 to 11.16 °Brix, with formulations containing orange peel extracts showing higher values. T6 had the highest TSS value at 11.16 °Brix, likely due to the combination of both extracts (Fig. 2 **(B)**). The lowest TA value (0.34 %) was found in T4, which contained 5 % *S. platensis* extract, while the highest TA value (0.50 %) was observed in T6, with T5 showing a similar value (0.48 %) (Fig. 2 **(C)**). The increased acidity content in the formulations containing orange peel extract likely contributed to the lower pH of T5 compared to the other formulations. Our findings are consistent with previous studies on functional products enriched with *S. platensis* and orange-derived ingredients. The study by Hassanzadeh et al. [4] on pear-cantaloupe juice reported that the addition of *S. platensis* did not significantly alter pH, TSS, or TA in the cantaloupe and pear juice blend, aligning with our observations. Similarly, research by Acharjee et al. [58] demonstrated results comparable to ours, showing that the inclusion of orange pomace powder in yogurt increased TSS and TA while reducing pH. Additionally, El-Nawasany et al. [51] confirmed that the addition of orange peel powder increased TSS and TA while reducing pH, further supporting our findings.

### 3.5 Total phenolic content (TPC), total flavonoid content (TFC) and total carotene content (TCC) of formulations

**Fig. 3:**
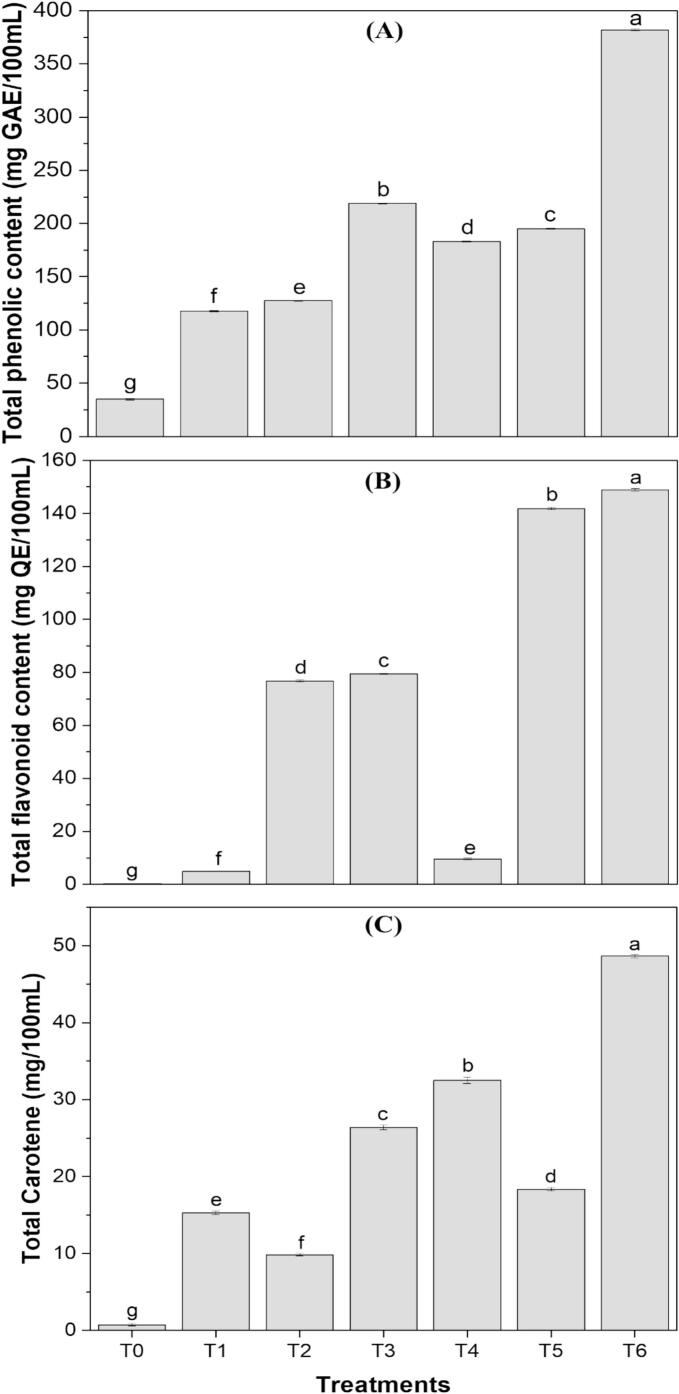
(A) Total phenolic content, (B) Total flavonoid content, and (C) Total carotene content of clean-label functional drink formulations (T1, T2, T3, T4, T5, T6). Treatments with different letters above the bars indicate significant differences (P < 0.05) between the formulations.

Bioactive compounds play a crucial role in promoting human health, driving consumer interest toward functional food products [56]. The TPC, TFC, and TCC of the formulations are presented in Fig. 3, showing a significant increase in bioactive compounds with the addition of active clean-label extracts in the juice blends making them a suitable functional drink. The control sample containing juice blends exhibited the lowest values for TPC (34.97 ± 0.70 mg GAE/100 mL), TFC (2.12 ± 0.06 mg QE/100 mL), and TCC (0.73 ± 0.25 mg/100 mL), whereas formulation T6, which contained highest concentrations of both extracts, showed the highest values for TPC (143.02 ± 0.66 mg GAE/100 mL), TFC (148.92 ± 0.51 mg QE/100 mL), and TCC (48.66 ± 0.22 mg/100 mL). This represents an approximately fourfold increase in TPC, a seventy-fold increase in TFC, and a sixty-six-fold increase in TCC. The continuous increase in TPC from the control to T6 can be attributed to the high phenolic content of both extracts. The different studies have also demonstrated a similar trend of increase in TPC in their products such as pasta [38], cookies [13], yogurt [10] and sports drinks [55] with the incorporation of algal extracts. However, TFC exhibited an even greater increase, primarily due to the orange peel extract, a rich source of flavonoids, while *S. platensis* contains only negligible amounts. Consequently, formulations containing orange peel extract displayed significantly higher TFC values compared to those containing algal extract. In a similar approach, El-Nawasany et al. [51] and El‐beltagi et al. [16] showed an increase in TPC and TFC of yogurt and cakes with the addition of orange peel. In the case of TCC, both extracts contributed to the increase in carotenoid content, but *S. platensis* extract, being particularly rich in carotenoids, resulted in higher TCC values in formulations containing this extract. In a study by Barkallah et al. [10] the TCC increased from 0 to 10 mg/g with the addition of *S. platensis* extract in yogurt and 0 to 31 mg/L with the addition of carrot pomace in milk [54].

### 3.6 Antioxidant activity of formulations

**Fig. 4:**
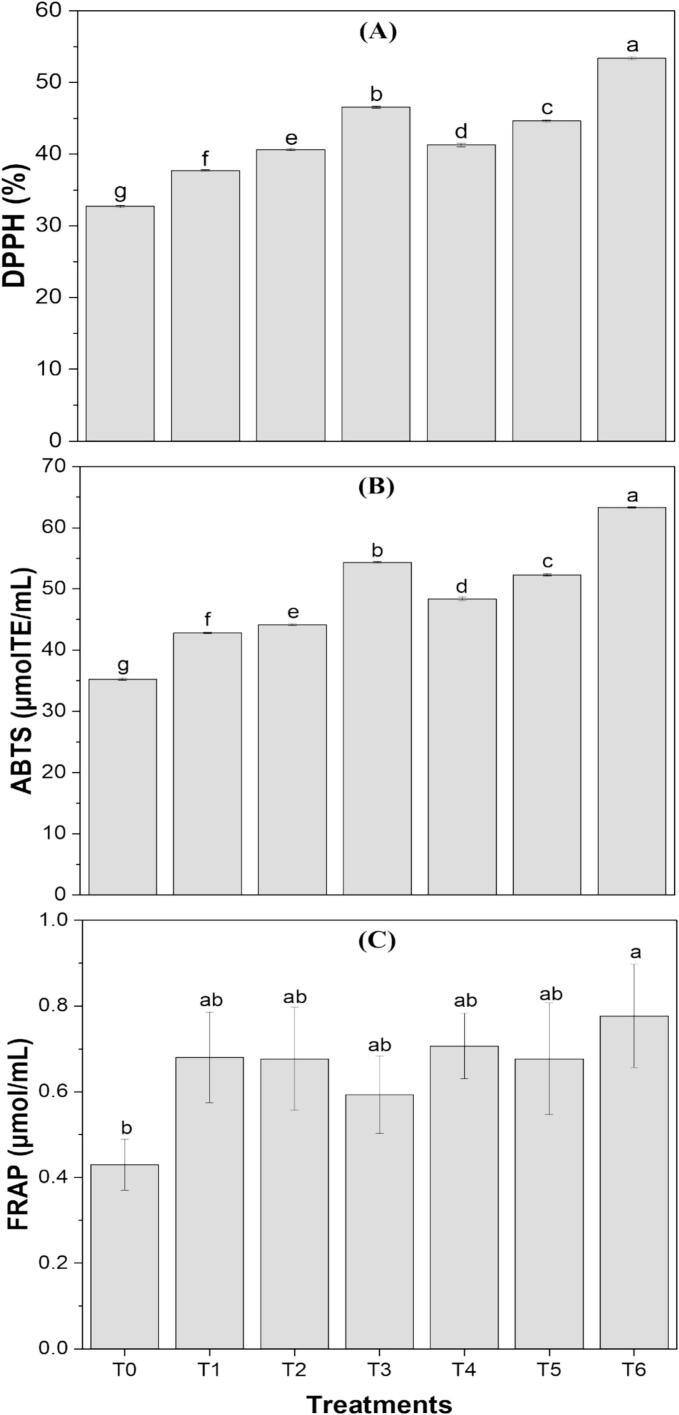
Antioxidant activity (A) DPPH (1,1-diphenyl-2-picrylhydrazyl) radical scavenging assay, (B) ABTS (2,2′-azino-bis(3-ethylbenzothiazoline-6-sulphonic acid)) assay, and (C) FRAP (Ferric Reducing Antioxidant Power) assay of clean-label functional drink formulations (T1, T2, T3, T4, T5, T6). Treatments with different letters above the bars indicate significant differences (P < 0.05) between the formulations.

Three different assays were conducted to evaluate the antioxidant activity of the formulations, and the results are presented in Fig. 4. The presence of bioactive compounds in the algal and waste extracts contributed to a significant increase in antioxidant activity in the formulations compared to the control [27]. The DPPH (%) inhibition was highest for T6 (58.26 ± 0.20 %) compared to the control (32.71 ± 0.20 %), indicating a substantial improvement in free radical scavenging activity by the incorporation of extracts. Similarly, the ABTS value for T6 was 8.34 ± 0.07 µMTE/mL, significantly higher than that of the control sample (3.25 ± 0.13 µMTE/mL). In the same way, FRAP antioxidant capacity of the control (5.42 ± 0.08 μM Fe^2+^/mL) was extremely lower than that of the T6 (16.74 ± 0.09 μM Fe^2+^/mL). Besides TPC and TFC of extracts, the chlorophyll and vitamins of *S. platensis* and orange peel extracts played a significant role in increasing the antioxidant activity [13]. The analogous findings have been documented by other researchers on increase in antioxidant activity by incorporation of algal and waste materials like Barkallah et al. [10] reported a 9 % increase in yogurt, Fradinho et al. [38] reported 20 % increase in cooked pasta, Hassanzadeh et al. [4] reported 10 % increase in pear-cantaloupe juice and El‐beltagi et al. [16] reported 3 % increase in cakes.

### 3.7 Reducing, non-reducing, total sugars and ascorbic acid of formulations

Sugars play a vital role in determining the sweetness, stability, and overall quality of functional beverages. The reducing, non-reducing, and total sugar content of the formulations are presented in Table 4. The results indicated that the addition of orange peel extracts led to an increase in sugar values, whereas *S. platensis* had a non-significant effect on sugar content. The control sample had lower values for reducing sugars (9.14 ± 0.03), non-reducing sugars (4.11 ± 0.07), and total sugars (13.11 ± 0.10) compared to formulations containing orange peel extracts, particularly T5 and T6, with 5 % extract. The sugar content in these formulations increased significantly, with T5 and T6 showing reducing sugar values of 9.93 ± 0.05 and 10.11 ± 0.07, non-reducing sugar values of 5.46 ± 0.05 and 5.59 ± 0.07, and total sugar values of 15.31 ± 0.06 and 15.49 ± 0.08, respectively. Strawberry and cantaloupe juices are naturally rich in ascorbic acid and the ascorbic acid content significantly increased with the incorporation of orange peel extracts in the drink formulations (Table 4). The control formulation (T0) had an ascorbic acid content of 34.19 ± 0.09, which increased to 37.12 ± 0.10 in T2, 40.34 ± 0.11 in T5, and 40.10 ± 0.09 in T6. This increase can be attributed to the presence of orange peel extract, which is naturally rich in ascorbic acid. On the other hand, the incorporation of *S. platensis* extract had a non-significant effect on the ascorbic acid content of the drink formulations. To the best of our knowledge, no studies have been conducted on the evaluation of changes in reducing sugars, non-reducing sugars, total sugars, and ascorbic acid in beverages formulated with algal or waste extracts. This study provides novel insights into the impact of these bioactive extracts on the sugar composition and nutritional quality of functional beverages, contributing to the advancement of research in this field.

**Table 4:** Reducing sugars, non-reducing sugars, total sugars, ascorbic acid, viscosity, cloud value, non-enzymatic browning, in-vitro digestibility and color parameters (L*, a*, b*) of clean-label functional drink formulations.

**Treatments**	**Reducing sugars** **(%)**	**Non-reducing sugars (%)**	**Total sugars** **(%)**	**Ascorbic Acid (mg/100 mL)**	**Viscosity (cp)**	**Cloud Value**	**Non-enzymatic browning**	**Invitro-digestibility (%)**	**Color values**		
									L*	a*	b*
**T0**	9.14 ± 0.03^c^	4.11 ± 0.07^c^	13.11 ± 0.10^c^	34.19 ± 0.09^c^	8.04 ± 0.06^f^	0.72 ± 0.04^b^	0.61 ± 0.05^a^	95.20 ± 0.35^a^	59.79 ± 0.08^a^	16.28 ± 0.07^e^	21.30 ± 0.08^e^
**T1**	9.18 ± 0.07^c^	4.17 ± 0.06^c^	13.33 ± 0.07^c^	34.21 ± 0.08^c^	8.49 ± 0.05^de^	0.74 ± 0.07^ab^	0.61 ± 0.03^a^	93.67 ± 0.25^bc^	51.29 ± 0.08^c^	12.57 ± 0.07^f^	16.72 ± 0.07^f^
**T2**	9.65 ± 0.06^b^	4.90 ± 0.08^b^	14.40 ± 0.07^b^	37.12 ± 0.10^b^	8.39 ± 0.07^e^	0.75 ± 0.05^ab^	0.63 ± 0.05^a^	94.58 ± 0.23^ab^	61.08 ± 0.06^a^	19.29 ± 0.06^c^	27.29 ± 0.09^b^
**T3**	9.78 ± 0.07^b^	4.98 ± 0.06^b^	14.72 ± 0.07^b^	37.11 ± 0.04^b^	8.93 ± 0.06^b^	0.78 ± 0.05^ab^	0.64 ± 0.04^a^	92.88 ± 0.50^c^	55.00 ± 0.09^b^	17.40 ± 0.07^d^	22.47 ± 0.11^d^
**T4**	9.29 ± 0.07^c^	4.27 ± 0.06^c^	13.55 ± 0.05^c^	34.20 ± 0.13^c^	8.80 ± 0.09^bc^	0.79 ± 0.06^ab^	0.62 ± 0.04^a^	91.06 ± 0.26^d^	42.21 ± 0.10^d^	7.73 ± 0.06^g^	13.83 ± 0.05^g^
**T5**	9.93 ± 0.05^a^	5.46 ± 0.05^a^	15.31 ± 0.06^a^	40.34 ± 0.11^a^	8.62 ± 0.07^cd^	0.79 ± 0.07^ab^	0.65 ± 0.05^a^	93.83 ± 0.21^b^	64.37 ± 0.09^a^	25.46 ± 0.08^a^	31.29 ± 0.06^a^
**T6**	10.11 ± 0.07^a^	5.59 ± 0.07^a^	15.49 ± 0.08^a^	40.10 ± 0.09^a^	9.25 ± 0.05^a^	0.84 ± 0.05^a^	0.66 ± 0.04^a^	89.57 ± 0.41^e^	59.31 ± 0.06^a^	19.92 ± 0.08^b^	24.37 ± 0.06^c^

Data are expressed as means ± SD. Values with different letters in each column are significantly different (P < 0.05).

### 3.8 Color of formulations

Color is a crucial quality parameter for beverages, as visual appearance is the first attribute perceived by consumers and significantly influences their purchasing decisions. The variation in color values of different formulations is presented in Table 4. The control (T0) had L*, a*, and b* values of 59.79 ± 0.08, 16.28 ± 0.07, and 21.30 ± 0.08, respectively. The L* value, which represents lightness, decreased with the increasing concentration of *S. platensis* but increased with the orange peel extracts due to their different pigment composition. The lowest L* value was recorded for T4 (42.21 ± 0.10), while T6, which contained both extracts, had an almost similar L* value (59.31 ± 0.06) with control, possibly due to the balancing effect of both extracts on color retention. The a* values, which represent redness (positive values) and greenness (negative values), showed a decreasing trend with the addition of *S. platensis* extract. The a* value reduced from 16.28 ± 0.07 in the control (T0) to 12.57 ± 0.07 in T1 and further to 7.73 ± 0.06 in T4 with the incorporation of 2.5 % and 5 % algal extracts, respectively. Conversely, the addition of orange peel extract increased the a* values, reaching 19.29 ± 0.06 in T2 and 25.46 ± 0.08 in T5. However, when both extracts were combined, the a* values remained relatively stable at 17.40 ± 0.07 in T3 and 19.92 ± 0.08^b^ in T6, suggesting a balancing effect between the two extracts on color modification. Similar to the L* and a* values, the b* values followed the same trend, increasing with the addition of orange peel extract and decreasing with the incorporation of *S. platensis* extract. A stabilizing effect was observed when both extracts were combined. The highest increase in b* value was seen with the addition of 5 % orange peel extract, rising from 21.30 ± 0.08 in T0 to 31.29 ± 0.06 in T5. Conversely, the greatest decrease was observed in T4, where the b* value dropped to 13.83 ± 0.05. In formulations containing both extracts, the b* values remained intermediate, with T3 and T6 showing values of 22.47 ± 0.11 and 24.37 ± 0.06, respectively. The decrease in color values of functional drink formulations with the addition of *S. platensis* is attributed to the high concentration of blue-green pigments chlorophylls and phycocyanin [59]. Our results are consistent with the studies of Barkallah et al. [10], Fradinho et al. [38], Batista et al. [13] and Rezvani & Goli [54] conducted on the incorporation of different concentrations of algal and waste materials in different food products and the potential changes in color values that occur.

### 3.9 Viscosity, cloud value, and non-enzymatic browning of formulations

Viscosity, cloud value, and non-enzymatic browning are key factors affecting the texture, stability, appearance, and shelf life of beverages. Optimizing viscosity ensures proper mouthfeel and cloud value maintains visual appeal and nutrient suspension, while controlled non-enzymatic browning preserves color, flavor, and nutritional quality. The values of these parameters for our formulations are shown in Table 4. The viscosity values showed minimal increase with the increasing concentration of extracts, particularly in formulations containing *S. platensis* extract. The viscosity ranged from 8.04 ± 0.06 in the control sample (T0) to 9.25 ± 0.05 in T6. The minor increase can be attributed to the presence of solids in the extracts, which contribute to the thickening effect of the beverage. The viscosity values fall within the ideal range, ensuring a balanced texture. Excessively high viscosity can hinder the flowability of the drink, making it difficult to pour and consume, while low viscosity may promote sedimentation, affecting the uniformity and stability of the beverage. Both extremes can negatively impact consumer acceptance, highlighting the importance of achieving an optimal viscosity for drinks. The analogous findings have been demonstrated by Elkot et al. [55] and El-Nawasany et al. [51] in their research on the increase in viscosity of sports beverages by *S. platensis* and yogurt by orange peel powder. A similar trend was observed in the cloud value of formulations, which increased from 0.72 ± 0.04 in T0 to 0.84 ± 0.04 in T6, where higher concentrations of extracts were present. This increase is attributed to the suspended solid particles composed of complex macromolecular mixtures. Higher cloud values indicate better stability, as lower values suggest sedimentation, which can negatively impact the visual appeal and consistency of the drink [54]. These results align with the Fatima et al. [7] where authors reported an increase in cloud value with the addition of different concentrations of various extracts. The non-enzymatic browning of the formulations showed non-significant values with the addition of extracts. The non-enzymatic browning is due to the formation and accumulation of brown compounds (phenolic compounds, pigments, and Maillard reaction precursors). The value for T0 was 0.61 ± 0.05, which increased to 0.63 ± 0.05 in T2 and 0.65 ± 0.05 in T5, indicating a minor rise due to the presence of orange peel extract. In contrast, the increase was negligible comparatively in formulations with *S. platensis* extract, with values of 0.61 ± 0.03 in T1 and 0.62 ± 0.04 in T4. The formulations containing both algal and waste extracts, T3 and T6, exhibited intermediate values of 0.64 ± 0.04 and 0.66 ± 0.04, respectively, suggesting a balanced effect of both extracts at different concentrations. It's likely possible that the extracts will reduce non-enzymatic browning during storage because of their composition. In a study, Chen et al. [60] reported a reduction in non-enzymatic browning of the orange juice during storage added with epigallocatechin gallate and l-cysteine. However, to the best of our knowledge, no studies have yet been conducted on evaluating the impact of algal and waste extracts on non-enzymatic browning in juice blend-based functional drinks.

### 3.10 In-vitro digestibility of formulations

The assessment of in-vitro digestibility of functional food products is essential to determine their behavior under simulated gastrointestinal conditions. The in-vitro digestibility results of these novel clean-label functional drink formulations are presented in Table 4. The highest digestibility was observed in the control formulation of juice blends only (T0) at 95.20 ± 0.35 %, while the lowest was in T6 (89.57 ± 0.41 %), which contained both extracts at 5 % concentration. The slight reduction in digestibility, particularly in T6, may be attributed to the presence of fibers, polyphenols, and protein-extract interactions. Formulations containing algal extracts exhibited slightly lower digestibility compared to those with waste extracts. Additionally, a higher concentration of bioactive compounds in the extracts contributed to the decrease in digestibility. To the best of our knowledge, no previous studies have investigated the in-vitro digestibility of functional beverages formulated with algal and waste extracts. This study provides novel insights into the potential effects of these bioactive ingredients on the digestibility of functional beverages. However, in a somewhat related study of Fradinho et al. [38] the authors reported a decrease in in-vitro digestibility of pasta with increasing concentration of *S. platensis.*

### 3.11 Microbial parameters of formulations

**Fig. 5:**
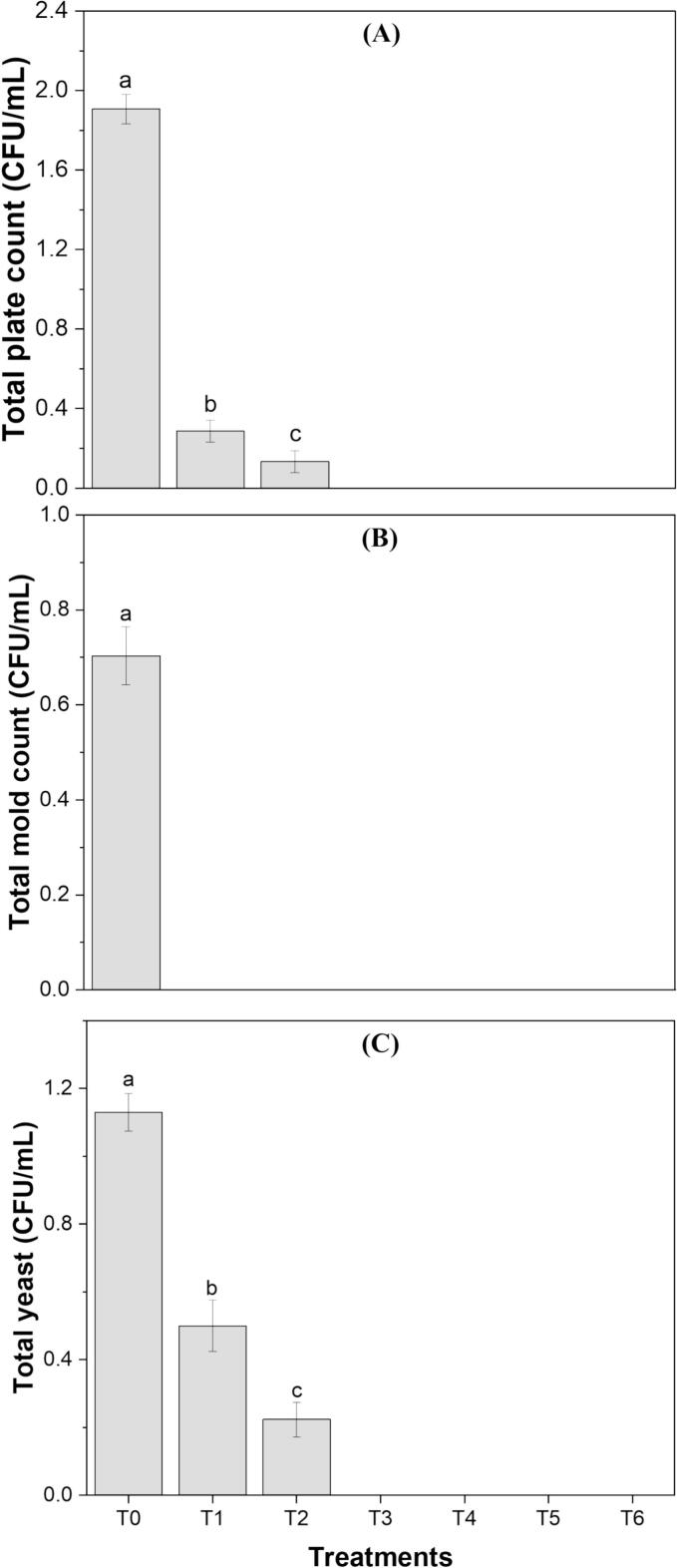
(A) Total plate count, (B) Total mold count, and (C) Total yeast count of clean-label functional drink formulations (T1, T2, T3, T4, T5, T6). Treatments with different letters above the bars indicate significant differences (P < 0.05) between the formulations.

The addition of *S. platensis* and orange peel extracts improves the microbial stability of the formulations due to their antimicrobial and antioxidant compounds as shown in Fig. 5. Many studies have been conducted on the use of natural extracts as preservatives in beverages, demonstrating exceptional results in their ability to enhance preservation. These natural extracts have shown significant antimicrobial, antioxidant, and shelf-life-extending properties, making them a promising alternative to synthetic preservatives [61]. The microbial analysis revealed that only the control sample (T0), which lacked any extracts, exhibited microbial presence, with a total plate count of 2.29 log CFU/mL, a total yeast count of 1.83 log CFU/mL, and a total mold count of < 1 log CFU/mL. In contrast, formulations containing 2.5 % and 5 % extracts showed low or no microbial growth, indicating the potent antimicrobial properties of the added extracts. Especially the formulations having combined algal and waste extracts showed no signs of microbes. However, the storage study of this functional drink can further determine its shelf life compared to the control juice blend. The extracts exhibited rapid antimicrobial activity by disrupting microbial cells and inhibiting their growth, primarily due to their rich bioactive composition. The presence of phenolic compounds, flavonoids, and other bioactive metabolites contributed to their strong antimicrobial potential, making them effective against a range of spoilage and pathogenic microorganisms [7]. Our findings align with Hassanzadeh et al. [4], Barkallah et al. [10], and El‐beltagi et al. [16] who reported that by the addition of algal and waste extracts in the food products, there were not any microbes detected.

### 3.12 Sensory properties of formulations

**Fig. 6:**
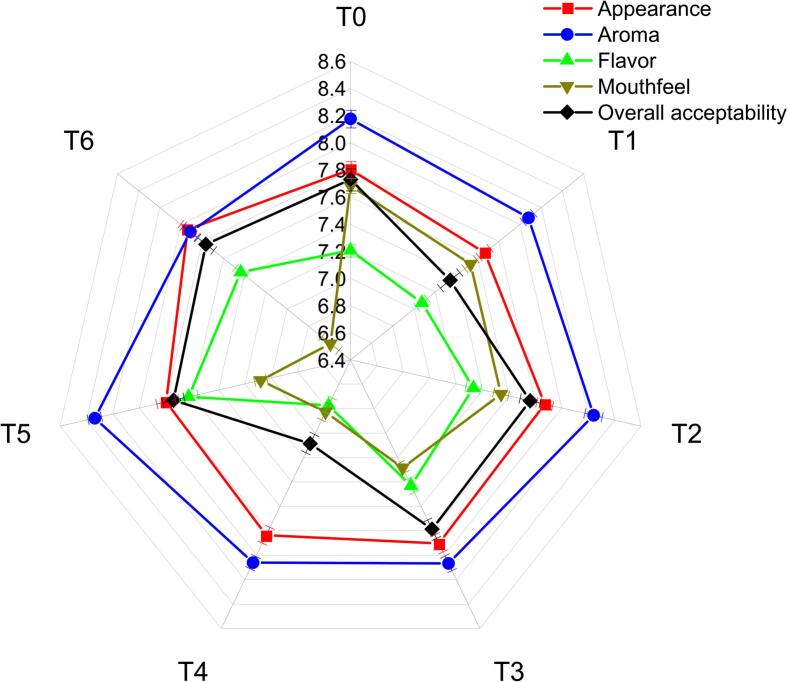
Sensory parameters (Appearance, Aroma, Flavor, Mouthfeel, Overall acceptability) of clean-label functional drink formulations (T1, T2, T3, T4, T5, T6).

Sensory evaluation is a critical factor in determining consumer acceptance of functional beverages. The results (Fig. 6) highlight variations in sensory parameters across different formulations, with the highest overall acceptability observed in T3 (7.78), followed by T6 (7.76) and the lowest observed in T4 (7.08) as compared to T0 (7.73). The appearance was most preferred in T6 (7.99), likely due to the color enhancement from both extracts, whereas T1 (7.66) had the lowest value. The aroma was significantly improved in T2 (8.24) and T5 (8.33), indicating the positive contribution of orange peel extracts, which contain volatile compounds that enhance the sensory profile. However, T4 had the lowest aroma score (7.75), possibly due to the strong pigment and aroma of *S. platensis*, which might not be as appealing to all consumers. The incorporation of extracts enhanced flavor perception, with T5 scoring the highest (7.62), slightly higher than T6 (7.43), and much better than the control (6.65). This suggests that the compounds in orange peel extract contributed positively to the flavor. However, the mouthfeel parameter was highest in the control formulation (7.68), while the lowest was observed for T6 (6.59), possibly due to the textural modifications caused by *S. platensis*, which has been reported to affect the viscosity and smoothness of food matrices. The strawberry-cantaloupe functional drink exhibits high sensory appeal in terms of appearance, aroma, flavor, mouthfeel, and overall acceptability. Both fruits were chosen due to their natural sweetness, vibrant color, and exceptional aroma, making them ideal for developing a superior functional beverage. Their blend not only enhances sensory properties but also creates a novel, visually appealing color that stimulates thirst. The unique combination produces an unmatched aroma, and unique mouthfeel, unlike any other drink, while the naturally sweet flavor eliminates the need for added sugars or sweeteners. These attributes contribute to high consumer preference and acceptance as shown by results, making it a standout functional drink. The findings of our study align with previous literature. A study by Fradinho et al. [38] reported similar results in sensory parameters of pasta incorporated with *A. platensis*. Similarly, El-Nawasany et al. [51] demonstrated an enhancement in sensory attributes of yogurt by adding orange peel powder, which is consistent with our findings. Overall, our study suggested that strawberry and cantaloupe-based functional drinks provide a well-accepted formulation, with orange peel extract enhancing aroma and flavor while *S. platensis* slightly influences mouthfeel. Despite minor variations in individual sensory parameters, the combination of both extracts in T3 resulted in the highest overall acceptability slightly more than in T6, demonstrating the potential of extracts combination in developing consumer-preferred functional beverages.

### 3.13 Principal component analysis (PCA)

**Fig. 7:**
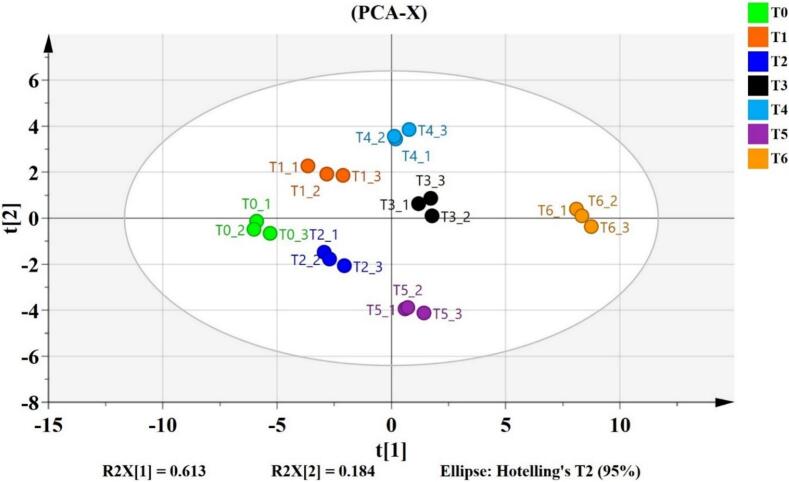
Principal component analysis (PCA) among clean-label functional drink formulations (T1, T2, T3, T4, T5, T6).

The PCA score plot (Fig. 7) reveals the distribution of formulations, with PC1 and PC2 explaining 79.7 % of the total variation. PC1 (61.3 %) primarily differentiates formulations based on extract concentrations and their impact on physicochemical properties such as color, viscosity, and browning. PC2 (18.4 %) captures secondary variations, likely linked to sensory attributes and microbial characteristics. Treatments separated by PC1 are more different than treatments separated by PC2. Moreover, the distinct separation of extract-containing formulations from the control indicates that *S. platensis* and orange peel extracts significantly influence the overall characteristics of the functional drinks.

## 4 Conclusion

This research addressed a critical challenge posed by shifting consumer trends toward healthy and sustainable products by developing a novel clean-label functional drink using sustainable ingredients, processes, and technologies. The drink was formulated using strawberry and cantaloupe juice blends, enriched with clean-label extracts of *S. platensis* and orange peel, obtained through a sustainable, innovative, and clean-label UAE with NADES method. These extracts were selected due to their superior composition compared to those obtained using conventional methods. Furthermore, the application of US assisted homogenization significantly improved the overall quality of the drink, enhancing its stability, texture, nutrient bioavailability, and sensory attributes. The experimental results demonstrated that incorporating these extracts significantly enhanced the drink’s proximate composition, mineral content, bioactive compounds, antioxidant activity, antimicrobial properties, and sensory profile. Among the various formulations tested, the T6 variant, which contained 5 % *S. platensis* and 5 % orange peel extract, yielded the best overall results across all parameters, although it exhibited slightly lower in-vitro digestibility compared to other formulations. This novel clean-label functional beverage represents a significant step forward in developing health-promoting drinks using multiple natural ingredients without relying on chemical additives or preservatives. The study emphasizes that carefully optimizing functional ingredients extracted through green and advanced techniques can maximize the nutritional value of foods and beverages while eliminating synthetic components, as natural extracts offer a broad spectrum of health benefits. Importantly, the drink was formulated with a high juice content (70 %), which is substantially higher than that found in most commercial products. The aim was to develop a beverage that delivers essential nutrients in a single serving, reduces disease risk, and supports overall well-being. However, further research is warranted to conduct storage studies for shelf-life determination, evaluate thermal and non-thermal preservation techniques to ensure long-term stability without compromising nutritional integrity, and perform in-vivo studies to assess the health impact of this drink. Future studies could also explore the use of encapsulated, microencapsulated, or nanoencapsulated forms of *S. platensis* and orange peel extracts to further enhance their properties. Additionally, market analyses should be conducted to assess consumer acceptance and the commercial potential of this product, considering its promising sensory evaluation outcomes. In conclusion, this study lays a strong foundation for the development of innovative, sustainable, and functional beverages that meet modern consumer demands for health, clean labels, and natural ingredients while opening new avenues for future research and commercial application.

## 5 Compliance with ethical standards

Research involving human participants and/or animals

Sensory analysis involving human participants was conducted following ethical guidelines. Informed consent was obtained from all participants prior to the evaluation.

## 6 Declaration of generative AI in scientific writing

During the preparation of this work, the authors used ChatGPT to improve readability and language. After using this tool, the authors reviewed and edited the content as needed and they accept full responsibility for the content of the publication.

## CRediT authorship contribution statement

**Kashmala Chaudhary:** Writing – original draft, Visualization, Methodology, Formal analysis, Data curation, Conceptualization. **Samran Khalid:** Writing – review & editing, Writing – original draft, Methodology, Formal analysis, Data curation, Conceptualization. **Taghrid S. Alomar:** Writing – review & editing. **Najla AlMasoud:** Writing – review & editing. **Sadia Ansar:** Writing – review & editing. **Ahmed Fathy Ghazal:** Writing – review & editing, Visualization. **Abderrahmane Aït-Kaddour:** Writing – review & editing, Funding acquisition. **Rana Muhammad Aadil:** Writing – review & editing, Supervision, Methodology, Funding acquisition, Formal analysis, Data curation, Conceptualization.

## Declaration of competing interest

The authors declare that they have no known competing financial interests or personal relationships that could have appeared to influence the work reported in this paper.
